# Rosiglitazone metformin adduct inhibits hepatocellular carcinoma proliferation via activation of AMPK/p21 pathway

**DOI:** 10.1186/s12935-019-0732-2

**Published:** 2019-01-11

**Authors:** Yuyang Liu, Xiangnan Hu, Xuefeng Shan, Ke Chen, Hua Tang

**Affiliations:** 1 Key Laboratory of Molecular Biology for Infectious Diseases (Ministry of Education), Institute for Viral Hepatitis, Department of Infectious Diseases, The Second Affiliated Hospital, Chongqing Medical University, 1 Yi Xue Yuan Road, Chongqing, 400016 China; 2 Key Laboratory of Biochemistry and Molecular Pharmacology, Chongqing Medical University, Chongqing, China; 3 Department of Pharmacy, The First Affiliated Hospital of Chongqing Medical University, Chongqing, China

**Keywords:** Rosiglitazone metformin adduct, Hepatocellular carcinoma, Proliferation, Metformin, AMPK/p21 pathway

## Abstract

**Background:**

Rosiglitazone metformin adduct (RZM) is a novel compound, synthesized from rosiglitazone (Ros) and metformin (Met) combined at a molar mass ratio of 1:1. Met and Ros are widely used together for treating type 2 diabetes to improve drug effectiveness and reduce adverse drug reactions. Recent studies reported that both Met and Ros may possess antineoplastic properties in several cancers, including hepatocellular carcinoma (HCC). However, the effects of RZM in HCC and its underlying mechanisms remain unknown.

**Methods:**

RZM was synthesized from Ros and Met at an equal molar ratio and identified by infrared spectroscopy. MTS and colony formation assays were performed to detect proliferative repression of RZM, the mixture, Met and Ros, respectively. Tumorigenesis assay in vivo was used to confirm the anti-tumorigenesis potential of RZM and Met. Moreover, cellular apoptosis caused by RZM was analyzed by hoechst staining assay and flow cytometry. RT-qPCR and western blotting were performed to reveal mechanisms for the function of RZM.

**Results:**

Both in vitro and in vivo data showed that low doses of RZM enhanced inhibitory effect on HCC cells growth compared with Met. Flow cytometry analysis confirmed that treatment with RZM at 1 mM for 48 h triggered HCC cells apoptosis. RT-qPCR and western blotting analyses showed that p21 was upregulated in response to 1 mM RZM treatment. Furthermore, RZM could increase AMPK activation compared with Met. The increased p21 expression induced by RZM treatment was attenuated by an AMPK inhibitor compound C.

**Conclusions:**

All these observations demonstrate that RZM increases the antiproliferative effect of Met in HCC via upregulating p21 expression in an AMPK-dependent manner. Our results suggest that RZM has the potential to be an adjuvant for HCC therapy.

**Electronic supplementary material:**

The online version of this article (10.1186/s12935-019-0732-2) contains supplementary material, which is available to authorized users.

## Background

Diabetes mellitus (DM) is a group of metabolic disorders characterized by hyperglycemia due to defects in insulin secretion, insulin action, or both [1]. In recent decades, type 2 diabetes mellitus (T2DM) has rapidly become a global epidemic accompanied by lifestyle changes [2–4]. T2DM seriously threaten human health based on its various complications and increased risk of mortality [5, 6]. Biguanides and thiazolidinediones are regarded as current oral anti-diabetic drugs [7–9]. These two drugs alleviate hyperglycemia in complementary mechanisms. Metformin, a biguanide derivative, mainly increases the sensitivity of peripheral tissues to insulin and inhibits hepatic gluconeogenesis but has no effects on insulin. Rosiglitazone, which is representative of thiazolidinediones drugs, can improve insulin resistance and stimulate insulin secretion. However, a few diabetic patients given with Ros caused adverse drug reactions, such as the risk of cardiovascular and fracture events [10, 11]. Therefore, these two drugs are often jointly used in clinic to improve effectiveness and reduce side effects [8, 12, 13]. Recently, emerging epidemiological studies demonstrated that both Met and Ros could reduce the incidence of cancer and improve cancer prognosis in T2DM patients, such as hepatocellular carcinoma (HCC), breast cancer, colorectal cancer, pancreatic cancer, and esophageal cancer [14–17]. Since liver is an essential organ for energy and metabolism regulation, which is closely related to blood glucose control. Primary liver cancer, which consists mainly of HCC, is one of the most common malignant tumors worldwide [18]. Investigation on the protective effects and underlying mechanisms by these two drugs or adducts may provide a prospectively therapeutic approach for HCC treatment.

Here, we synthesized a new compound, RZM from Ros and Met combined at a molar mass ratio of 1:1. To determine whether RZM is able to inhibit HCC cells proliferation, we compared the antiproliferative effects of RZM, Ros and/or Met on HCC cells and explored the possible mechanisms.

## Materials and methods

### Materials and drug preparations

Rosiglitazone, metformin and compound C were purchased from Gao Meng Chemical Co., Ltd. (Beijing, China), Zhong Xin Pharmaceutical Co., Ltd. (Tianjin, China) and Apexbio Co., Ltd (USA), respectively. All these agents were dissolved in dimethyl sulfoxide (DMSO, Sigma, USA) and then diluted to indicated concentrations by phosphate-buffered saline (PBS, final concentration of DMSO < 0.1%) in vitro and in vivo experiments.

### Detection of infrared spectrum

Mortars and pestles were previously washed and placed in a dryer for 30 min. Then, each compound (1 mg) was ground into powder with dried KBr (50 mg) in a mortar. The mixture was pressed into a piece of slice and tested in an infrared spectrometer (Nicolet, USA).

### Cell culture

Human hepatoma cell lines HepG2, SK-hep1 and immortalized normal human liver cell line (L02) were obtained from the Chinese Academy of Sciences Cell Bank. Cells were cultured in DMEM (Gibco, USA) supplemented with 10% fetal bovine serum (FBS) and 1% penicillin/streptomycin under 37 °C and 5% CO_2_ humidified condition. Logarithmically growing cells were treated with indicated concentrations of each compound for 48 h and then replaced with regular culture medium.

### MTS assay

The effect on cellular proliferation was evaluated using Cell Titer96^®^ Aqueous One Solution Cell Proliferation Assay Kit (Promega, USA). This kit contains an MTS reagent, which was composed of [3-(4,5-dimethylthiazol-2-yl)-5-(3-carboxymethoxyphenyl)-2-(4-sulfophenyl)-2*H*-tetrazolium] and phenazinemethosulfate (PMS). 2 × 10^3^ cells were plated into each well of 96-well plates and incubated in regular culture medium for 6 h. Then, cells were treated with indicated doses of the agents for 48 h. 20 μL MTS reagents were added into each well and incubated at 37 °C for 2 h. The absorbance was measured at 490 nm. Experiments were performed in triplicate.

### Colony formation assay

5 × 10^2^ cells were seeded into six-well plates with indicated compounds treatment for 48 h and replaced with regular culture to continuously incubate for 10 days. Colonies were washed and fixed with 4% paraformaldehyde for 30 min. Then, colonies were stained with 0.1% crystal violet for 30 min and counted.

### Hoechst staining assay

Cells were plated at 5 × 10^4^ per well in 24-well plates. After treated for 48 h, cells were washed twice with PBS, fixed with 4% paraformaldehyde for 30 min and stained with 10 μg/mL hoechst reagent (Hoechst 33342, Beytime, China) for 15 min. Cells were then washed thrice for scanning at 200× by fluorescent microscope.

### Flow cytometric analysis of apoptosis

After treated with different compounds for 48 h, cells were harvested and washed in cold PBS, centrifuged thrice at 1000×*g* for 5 min and then resuspended in PBS. The assay using double staining (propidium iodide and annexin V) was analyzed by flow cytometry from Academy of Life Sciences (Chongqing Medical University, China).

### Reverse transcription and quantitative real-time PCR (RT-qPCR)

Total RNA was extracted from cells using Trizol reagent (Life Technologies Corporation, USA). RNA reverse transcription was processed following the manufacturer’s instructions by using a Reverse Transcription Kit (Takara, Japan). Then, RT-qPCR was performed using Faststart Essential DNA Green Master (Roche, Indianapolis, IN, USA). GAPDH was used as an internal control. All results expressed as the mean ± SEM were performed at least three independent experiments. Comparative quantification was determined using the 2^−ΔΔCt^ method. The premier sequences were as follows: p53 (forward): 5′-GGAAATTTGCGTGTGGAGTATTT-3′, (reverse): 5′-GTTGTAGTGGATGGTGGTACAG-3′; CCND1 (forward): 5′-CCTCGGTGTCCTACTTCAAATG-3′, (reverse): 5′-CACTTCTGTTCCTCGCAGAC-3′; p21 (forward): 5′-GTCACTGTCTTGTACCCTTGTG-3′, (reverse): 5′-TTTCTACCACTCCAAACGCC-3′; CDK2 (forward): 5′-AGATGGACGGAGCTTGTTATC-3′, (reverse): 5′-CTTGGTCACATCCTGGAAGAA-3′; CDK6 (forward): 5′-TCACGAACAG ACAGAGAAACC-3′, (reverse): 5′-CTCCAGGCTCTGGAACTTTATC-3′; EGFR (forward): 5′-G GAAGTACAAAGAGGAGGAAGAG-3′, (reverse): 5′-GGGAAGATGCCAGGGATAAA-3′; GADD45A (forward): 5′-GGAGAGCAGAAGACCGAAAG-3′, (reverse): 5′-GATCAGGGTAGTGGATCTG-3′; NANOG (forward): 5′-TCCTGAACCTCAGCTACAAAC-3′, (reverse): 5′-GCGTCACACCATTGCTATTC-3′; MYC (forward): 5′-GCTGCTTAGACGCTGGATTT-3′, (reverse): 5′ GAGTCGTAGTCGAGGTCATAGTT-3′; GAPDH (forward): 5′-CGGAGTCAACGGATTTGGTCGTAT-3′, (reverse): 5′-AGCCTTCTCCATGGTGGTGAAGAC-3′.

### Transient transfection of shRNA

Transfection of HCC cells with shRNA was carried out using Lipofectamine 2000 (Invitrogen, USA), according to the manufacturer’s instructions. Cells were grown to 50–70% density in 6-well plates before transfection. 2.5 μg sh-p21 or shNC was incubated for 6 h plus 5 µL lipofectamine reagent in Opti-MEM medium. Then, transfection medium was replaced by fresh regular culture medium. After 48 h of incubation, transfection efficacy was assessed by RT-qPCR and western blot analysis. The sequences of the RNA used in transfection were as follows: sh-p21 (forward): 5′-GATCCGGCTGATCTTCTCCAAGAGGACTTCCTGTCAGATCCTCTTGGAGAAGATCAGCCTTTTTG-3′, (reverse): 5′-AATTCAAAAAGGCTGATCTTCTCCAAGAGGATCTGACAGGAAGTCCTCTTGGAGAAGATCAGCCG-3′. sh-NC (forward): 5′-GATCCGCCACTTTGAAGAACCCAATCCTTCCTGTCAGAGATTGGGTTCTTCAAAGTGGCTTTTTG-3′, (reverse): 5′-AATTCAAAAAGCCACTTTGAAGAACCCAATCTCTGACAGGAAGGATTGGGTTCTTCAAAGTGGCG -3′.

### Protein extraction and western blot analysis

Cells were washed thrice with cold PBS and lysated in 100 μL RIPA lysis reagent (Ding Guo Biotechnology Co., Ltd, China) added 2 μL PMSF and 2 μL phosphatase inhibitor (Ding Guo Biotechnology Co., Ltd, China) for 30 min. Cells debris was removed by centrifugation at 14,000×*g* for 20 min at 4 °C. Protein concentration was measured by BCA assay (Ding Guo Biotechnology Co., Ltd, China). Clarified proteins lysated from each experimental condition (50 μg) were boiled for 5 min. Protein samples were subjected to 10% SDS-polyacrylamide gel electrophoresis (SDS-PAGE) and transferred onto polyvinylidene difluoride membrane (GE Healthcare life science, USA). After blocked with 5% non-fat milk in 1× TBST containing 0.05% Tween-20 for 2 h at room temperature, the membranes were incubated with specific primary antibodies overnight at 4 °C. Anti-mouse IgG and anti-rabbit IgG were used as secondary antibodies (Biosharp, China). Detection of specific proteins was performed by enhanced chemiluminescence reagent (ECL, Advansta, USA). The antibodies were as follows: anti-AMPKα (ref #ab32047, 1:3000, Abcam), Anti-phospho-specific (Thr172) AMPKα (ref #ab133448, 1:5000, Abcam), Anti-p21 (ref #ab109520, 1:2000, Abcam) and Anti-GAPDH (ref #10494-1, 1:5000, Bioworld).

### In vivo tumorigenesis assay

Four week-old male BALB/c-nude mice (purchased from Laboratory Animal Services Center of Chongqing Medical University) were randomly divided into three groups: RZM-treated group, Met-treated group and NC group. Mice were maintained under pathogen free conditions. All procedures for the mouse experiments were approved by the Animal Care Committee of Chongqing Medical College. HepG2 cells (1 × 10^7^, 200 μL) were subcutaneously injected into the left hind leg of the nude mice. Treatment was started when subcutaneous lesions were macroscopically apparent, reaching 30–50 mm^3^. Mice were treated with the agents by intraperitoneal injection (125 mg/kg), or sterilized saline as control every 4 days. Meanwhile, tumor size and mice weight were evaluated. Tumor volumes (expressed in mm^3^) were calculated using the following formula: 0.5 × (length × width^2^). Mice were sacrificed after 4 weeks of treatment. Subcutaneous tumor tissues were harvested and frozen in liquid nitrogen for protein analysis.

### Statistical analysis

The data from individual experiments are presented as the mean ± SD. Statistical comparisons between groups were done using one-way ANOVA followed by Dunnett post hoc testing. *P *< 0.05 was considered statistically significant using SPSS 22.0.

## Results

### Synthesis of rosiglitazone metformin adduct

**Fig. 1:**
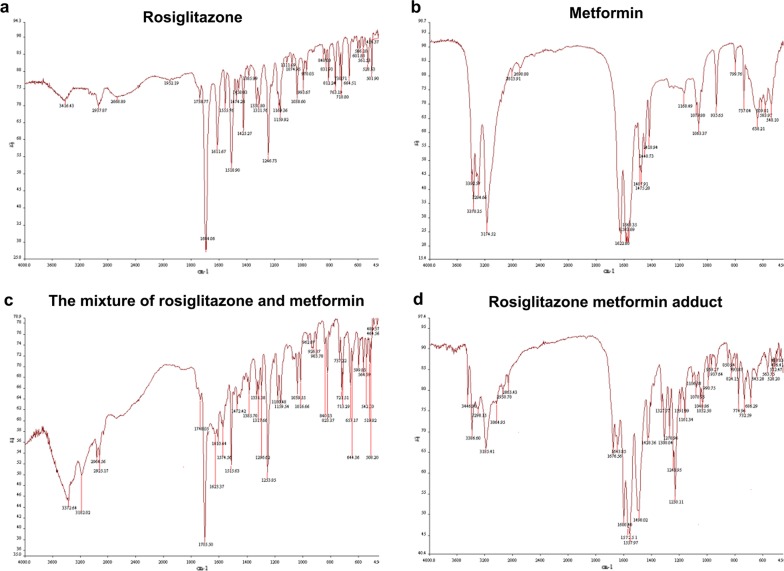
Synthesis of rosiglitazone metformin adduct (RZM). **a**–**d** The infrared spectroscopy analysis results of Ros, Met, the mixture of Ros and Met, and RZM

Rosiglitazone sodium (6 mmol), metformin (6 mmol) and absolute ethanol (120 mL) were collected in a round bottom flask. Then, the mixture was heated for 10 min under the condition of continuously whisking. After filtration, the liquid was distillated for removal of ethanol with a rotating evaporator and cooled down. At last, rosiglitazone metformin adduct (RZM) was obtained after recrystallized from absolute ethanol (2.26 g, yield 77%, melting point: 182–184 °C) [19]. The structure of Ros, Met, the mixture and RZM were identified with IR spectrum analysis and the data for characteristic absorption peaks were shown in Table 1. Ros possessed C=O bonds in thiolactone and lactam, aromatic oxide, =C–H bonds in benzene and pyridine ring and N–H bond (absorption peaks at 1738.37, 1694.06, 1246.73, 812.24, 763.19, 3416.43 cm^−1^) (Fig. 1a). Met possessed C=NH, N–H bond, N–H bending vibration, dimethyl anti-symmetric vibration, dimethyl symmetric vibration and NH_2_ bond (absorption peaks at 1622.80, 3392.57, 1583.69, 1568.35, 1487.91, 1475.28, 1448.73, 1418.94, 3294.66 cm^−1^) (Fig. 1b). Because Met has less mass fraction of the mixture at an equal molar mass ratio [MW: 166/(166 + 357)], a few peaks of Met were not obviously found in spectrum of the mixture. The results showed that absorption peaks of Ros and Met did not move and no new characteristic peaks were generated in IR spectrum of the mixture (Fig. 1c). However, the IR spectrum of RZM (Fig. 1d) indicated that N–H, C=O bonds of Ros, and NH_2_ of Met moved toward low-frequency vibration in RZM and generated sharp peaks at 3386.60, 1676.56, 1643.85, 3185.41 cm^−1^. These data confirmed that new hydrogen bonds were produced. At the same time, peaks of C=NH, N–H bending vibration, aromatic oxide, and =C–H bonds did not shift, suggesting that these functional groups did not participate in hydrogen bond formation or other chemical changes.

**Table 1:** Infrared spectrum analyses for Ros, Met, the mixture of Ros and Met, RZM

Chemical bonds and functional groups	Absorption peaks (cm^−1^)
Rosiglitazone	
C=O bonds in thiolactone and lactam	1738.37, 1694.06
Aromatic oxide	1246.73
=C–H bonds in benzene and pyridine ring	812.24, 763.19
N–H bond	3416.43
Metformin	
C=NH bond	1622.80
N–H bond	3392.57
N–H bending vibration	1583.69, 1568.35
Dimethyl anti-symmetric vibration	1487.91, 1475.28
Dimethyl symmetric vibration	1448.73, 1418.94
NH_2_ bond	3294.66
The mixture of rosiglitazone and metformin	
N–H bond	3372.64
NH_2_ bond	3182.02
C=O bonds in thiolactone and lactam	1748.05, 1705.50
Aromatic oxide	1253.85
=C–H bonds in benzene and pyridine ring	823.37, 721.51
C=NH bond	1623.37
Rosiglitazone metformin adduct	
C=O bonds in thiolactone and lactam	1676.56, 1643.85
C=NH bond	1600.40
NH_2_ bond	3185.41
N–H bond	3386.60
N–H bending vibration	1572.51, 1557.97
Aromatic oxide	1243.95
=C–H bonds in benzene and pyridine ring	824.15, 774.06

All above data showed that RZM is a novel adduct consist of Ros and Met, and these two agents were integrated by hydrogen bonds.

### The effects of RZM on HCC cells proliferation and apoptosis

**Fig. 2:**
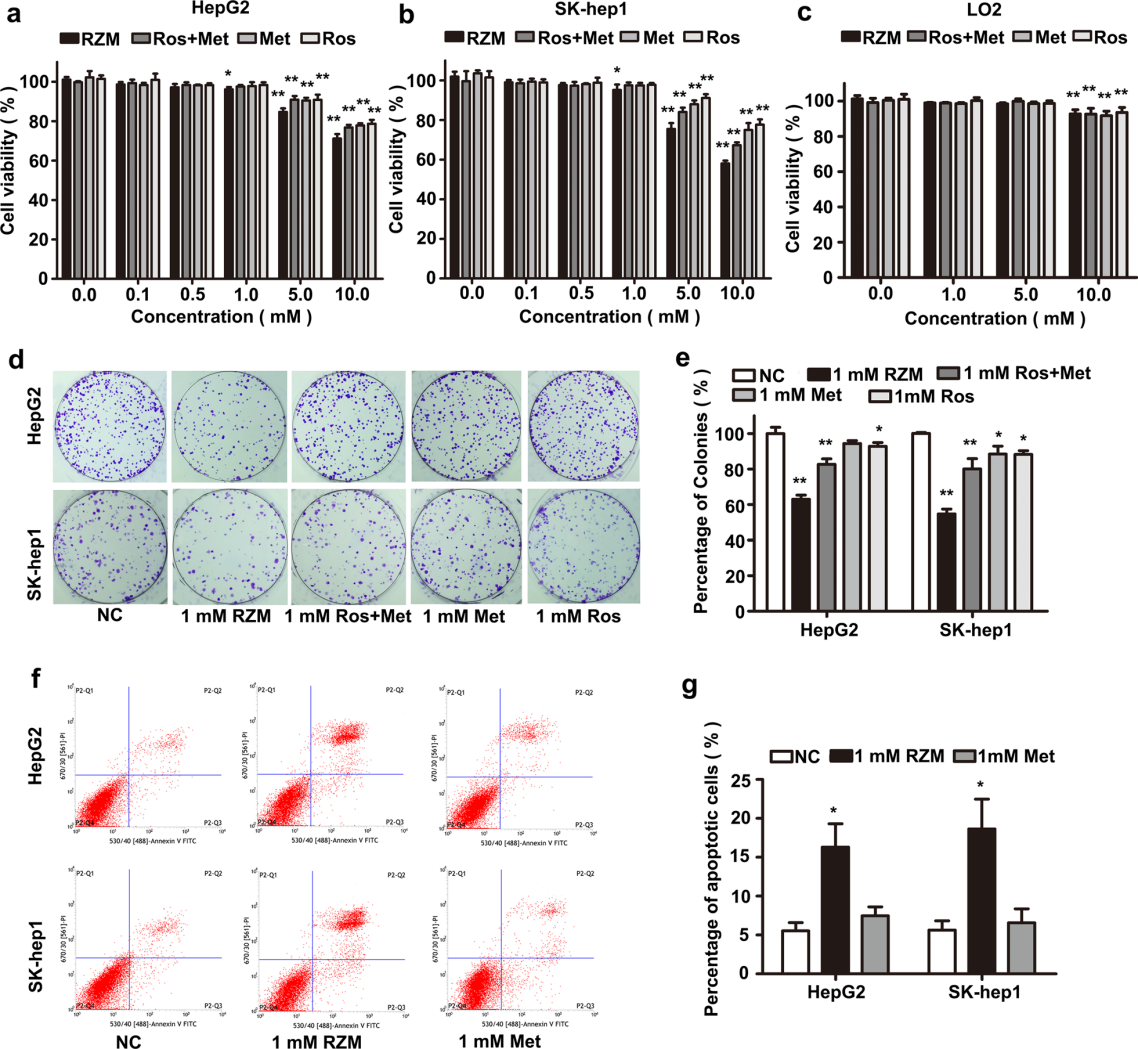
Effects of RZM, Ros and/or Met on cellular proliferation. **a**–**c** The antiproliferative effects induced by RZM, the mixture of Ros and Met, Ros and Met in HepG2, SK-Hep1 and L02 cells were detected by MTS assay (*P < 0.05 and **P < 0.01 versus the control group). **d** Representative images of colony formation of differently treated HCC cells were shown. **e** Quantitative results of colony formation assays were displayed (*P < 0.05 and **P < 0.01 versus the control group). All values were expressed as the means mean ± SD from at least three independent experiments. **f** HepG2 cells were treated with 1 mM RZM or Met for 48 h, flow cytometric analysis was used to determine the effects of the two compounds on cellular apoptosis. The apoptotic rates were represented by the ratios of early and late apoptotic cells (P2-Q3% + P2-Q2%). **g** Quantitative results of flow cytometric analysis were displayed (*P < 0.05 versus the control group)

MTS assay was performed to investigate the effect of RZM on HCC cells proliferation. We found that RZM inhibited two human hepatoma cells (HepG2 and SK-Hep1 cells) proliferation in a dose-dependent manner. Ros and/or Met also caused a dose-dependent inhibition in HCC cells. However, a significant reduction of proliferation was found in the presence of RZM at 1 mM, but not in other two agents (Fig. 2a, b). No significant difference was observed when normal human liver cell line L02 cells treated with low doses of RZM (Fig. 2c). Colony formation assay supported the results that RZM significantly decreased the percentage of colonies in HepG2 and SK-Hep1 cells compared with Ros and/or Met (Fig. 2d, e). These results indicated that low concentrations of RZM enhanced antiproliferative effects in HCC cells compared with Ros and/or Met, which did not impair the normal human liver cells viability. We further compared the apoptosis effects of RZM and Met on HepG2 cells. Results displayed that 1 mM Met did not cause cellular apoptosis, which is consistent with previous observations [20]. However, cells exposed to RZM at 1 mM for 48 h displayed cellular apoptosis with nuclear condensation by hoechst staining (Additional file 1: Figure S1). Furthermore, flow cytometric analysis was used to determine the effects of the two compounds on cellular apoptosis. Results showed that RZM triggered significantly cellular apoptosis at 1 mM, which was not found in Met-treated group (Fig. 2f, g). Taken together, the above results demonstrated that RZM inhibited HCC cells proliferation and caused apoptosis in vitro.

### The expression of p21 was upregulated in RZM treated HepG2 cells

**Fig. 3:**
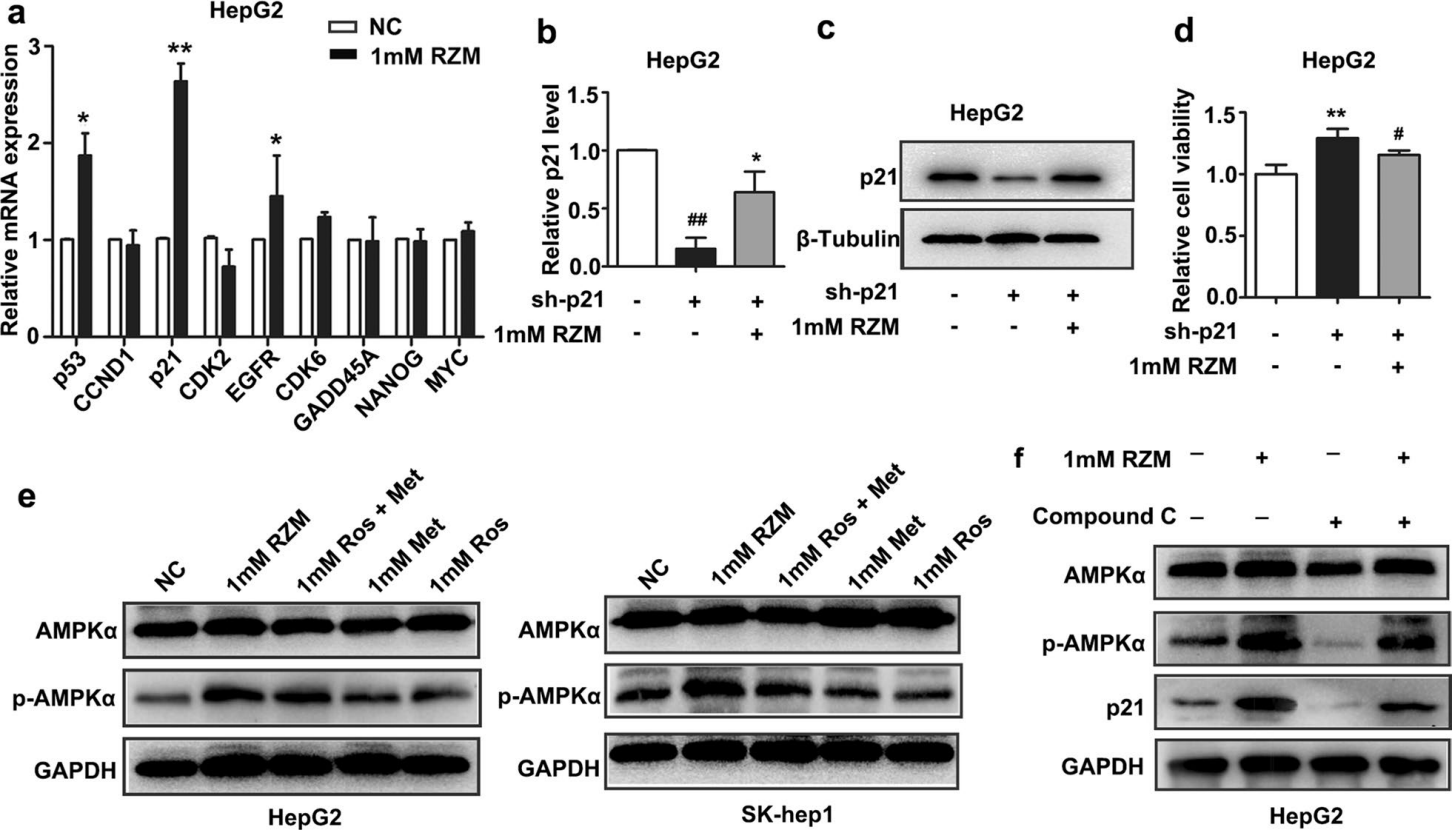
RZM upregulated the expression of p21 through the activation of AMPK. **a** RT-qPCR analysis of relative gene mRNA expressions in RZM-treated HepG2 cells. Transcript levels were normalized to GAPDH expressions (*P < 0.05 and **P < 0.01 versus the control group). **b**, **c** sh-p21 or/and RZM regulated the expression of p21 were assayed by RT-qPCR and western blotting assays (^##^*P *< 0.01 and *P < 0.05 versus the control group). **d** MTS assay conducted in HepG2 cells under the corresponding conditions. Values shown are mean ± SD of triplicate measurements (**P < 0.01 and ^#^*P *< 0.05 versus the control group). **e** Western blotting analysis of the activation of AMPK in HepG2 and SK-hep1 cells, using GAPDH as an endogenous control. **f** Relative protein expression of AMPK, p-AMPK and p21 in cells treated with RZM or/and compound C. GAPDH was used as an internal control

To explore the molecular mechanisms by which RZM repressed HCC proliferation, several crucial gene expressions relative to hepatocarcinogenesis were detected by RT-qPCR. As a result, the expression of p21 was obviously elevated in RZM-treated (1 mM) group compared with those of control group (Fig. 3a). Then, we constructed specific shRNA inhibiting p21 (sh-p21) and transfected into HepG2 cells. The knockdown efficiencies of p21 were confirmed on both mRNA and protein levels. Furthermore, the effects of p21 knockdown or/and RZM treatment on expressions of p21 were also detected. Results indicated that RZM at 1 mM partly reversed the depressed expression of p21 in sh-p21 transfected cells as well as restored HepG2 cells proliferation (Fig. 3b–d). In short, the above findings revealed that RZM could specifically upregulate the expression of p21.

### RZM modulated p21 expression via the activation of AMP-activated protein kinase

Previous studies have shown that the antineoplastic effects of Met might be mainly achieved by activation of AMP-activated protein kinase (AMPK) [21, 22]. Therefore, it was reasonable to hypothesize that RZM also activated AMPK. So we detected phosphorylation of AMPKα in HCC cells in response to these three agents by western blotting analysis. Results indicated that RZM enhanced the stimulatory effect on phosphorylation of AMPKα compared with Met and/or Ros (Fig. 3e). Compound C, an AMPK inhibitor, was used to demonstrate the role of AMPK in which RZM elevated the expression of p21. Results showed that compound C effectively alleviated increased expression of p21 induced by RZM, proving that RZM modulated p21 expression via the activation of AMPK (Fig. 3f). In all, the above results demonstrated that RZM enhanced HCC cells proliferation compared with Met via activation of AMPK/p21 pathway.

### RZM inhibited tumor growth in vivo

**Fig. 4:**
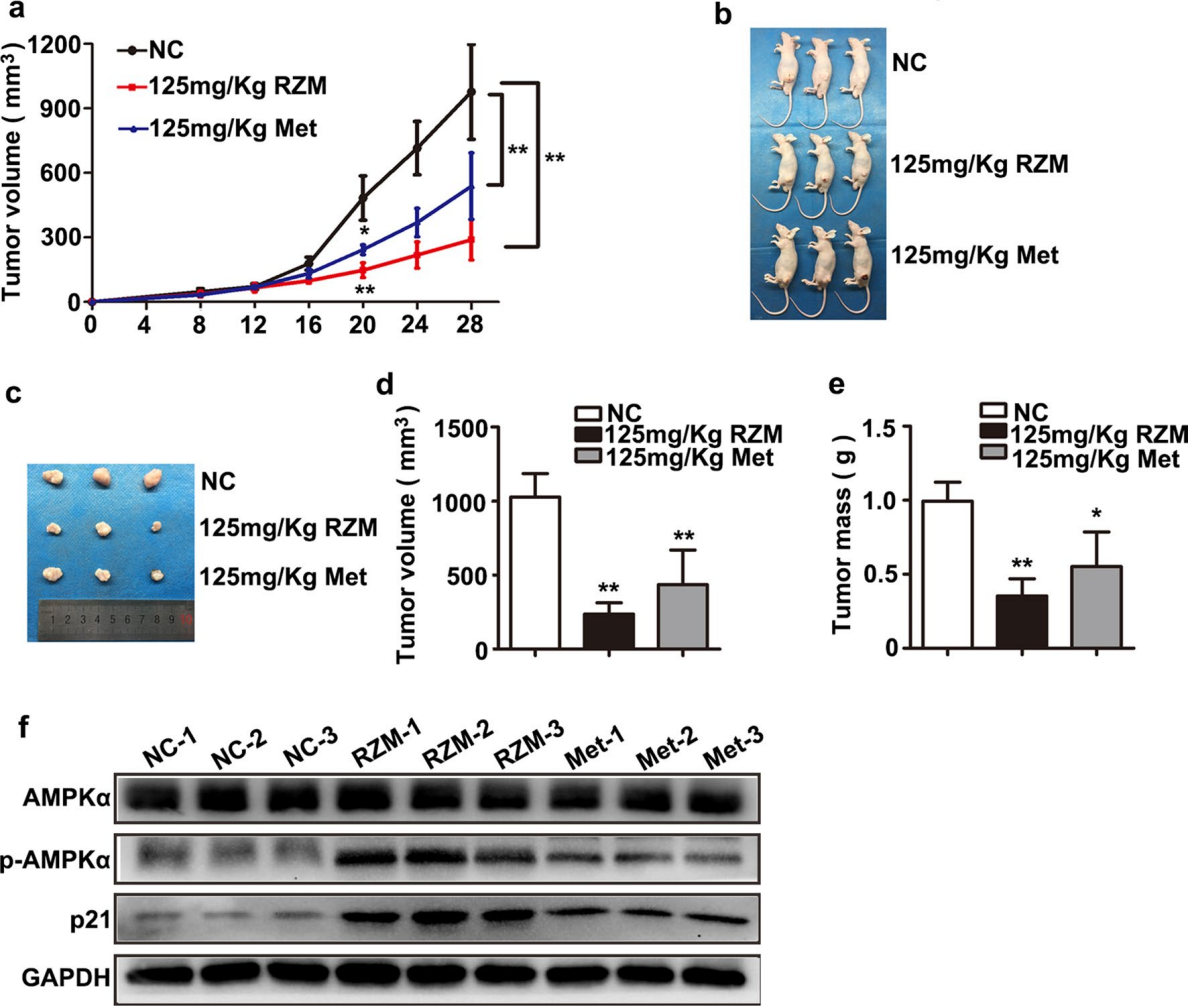
Effects of RZM and Met on tumor growth in vivo. **a** Tumor growth curves were analyzed for RZM-treated, Met-treated and the control group. (*P < 0.05, **P < 0.01 versus the control group). **b**, **c** Nude mice were sacrificed after 4 weeks and the tumors in each group were exhibited. **d**, **e** The mean volume and weight of xenograft tumors from indicated treatment were analyzed (*P < 0.05, **P < 0.01 versus the control group). **f** Relative protein expressions of AMPK, p-AMPK and p21 in RZM-treated, Met-treated and the control group (n = 3, each group)

Next, we investigated whether RZM could affect HCC tumor growth in vivo. HepG2 cells were injected into male nude mice to develop tumors. Treatment with the two compounds at 125 mg/kg or equal volume of sterilized saline was begun when the tumors were visible (the 8 day after implantation). The significant effects of two compounds on tumor growth were observed following 12 days of treatment (20 days after implantation; Fig. 4a). After 4 weeks, nude mice were sacrificed and the tumors in each group were exhibited (Fig. 4b, c). Both of two agents significantly suppressed tumor growth compared with the control group, while smaller tumor volumes and masses were observed in RZM treated mice (Fig. 4d, e). Moreover, the protein levels of p21 and p-AMPKα in tumor tissues were detected. Results showed that p21 and p-AMPKα expressions were obviously increased in RZM-treated group (Fig. 4f). During the course of treatment, the compounds did not cause noticeable side effects or changes in mice body weight (Additional file 2: Figure S2). Thus, we concluded that RZM could induce an additive inhibition on the tumor growth by increasing p21 and p-AMPK expressions in vivo.

## Discussion

Metformin, which is widely prescribed as an oral anti-diabetic agent, functions by increasing the insulin sensitivity of peripheral tissues, inhibiting gluconeogenesis and absorption by intestinal cells [23]. Since the drug effectively improves hyperglycemia with few side effects except for gastrointestinal intolerance and rare cases of lactic lactate acidosis [24], Met is recommended as a first-line drug for T2DM patients nowadays [23, 25]. However, Met is unable to improve insulin secretion and β-cell dysfunction, which can be complemented by another anti-diabetic drug Ros [26, 27]. As insulin sensitizers, Ros mainly acts on restoration of insulin secretion and reduction of insulin resistance [28, 29]. However, a series of severe adverse reactions occurred in Ros-treated T2DM patients, including obesity, osteoporosis, cardiovascular diseases and even heart failure [30–32]. Met and Ros are often jointly used in T2DM treatment for optimizing drug efficacy and reducing side effects [33, 34]. Interestingly, numerous of epidemiological studies and experiments have shown that both Met and Ros exerted antineoplastic abilities in certain cancers, including HCC. A population prospective cohort study based on 800,000 individuals indicated that there were great decreases of cancer incidence such as esophageal, liver, and pancreatic cancer in Met users [16]. Another study clarified that Ros exerted antineoplastic capacities in lung, prostate and colon cancer patients with T2DM [35]. Moreover, combination of Ros and Met were potentially related to improved survival in diabetic prostate cancer patients [36].

In our study, we synthesized a new compound, rosiglitazone metformin adduct (RZM) from Ros and Met combined at a molar mass ratio of 1:1. The results of infrared spectroscopy analysis revealed that the absorption peaks of the N–H, C=O bonds in Ros, NH2 bond in Met moved toward low-frequency vibration in RZM. These bonds were connected by hydrogen bonds. Results indicated that RZM was a new adduct rather than simply superposed. Next, we focused on exploring its actions and the possible mechanisms in HCC. AMP-activated protein kinase (AMPK) is a cellular energy sensor conserved in all eukaryotic cells, which plays a critical role in lipid and glucose metabolism. AMPK is a heterotrimeric protein complex formed by α, β, and γ subunits. AMPKα has catalytic activity, and it will be phosphorylated if AMPK is activated. The subunits of β and γ assist the maintenance of protein structure. Activation of AMPK normally occurs through a variety of receptors that increase the cellular AMP/ATP ratio. It has been reported that Met could activate AMPK in hepatocytes. Once activated, AMPK could regulate cell proliferation and metabolism via switches on catabolic pathways that allocate ATP [37, 38]. AMPK was also related to mediate its tumor suppression through regulation of p53 in HCC [21].

p21 is transcribed by cyclin dependent kinase inhibitor 1A (CDKN1A). The encoded protein acts as a tumor suppressor by inhibiting the activity of cyclin-dependent kinase 2 or cyclin-dependent kinase 4 complexes, resulting in cell cycle blocks at G1. Hundreds of studies demonstrated that p21 protein played an important role in cell cycle and DNA repair which is closely related to carcinogenesis [39]. The present study, we firstly evaluated the antiproliferative abilities of RZM both in vitro and in vivo experiments. In this setting, we elucidated that low concentrations of RZM enhanced HCC cells antiproliferative effect without affection of human normal cell line L02. We elucidated this action was owing to upregulation of p21 expression. Furthermore, a more significantly elevated phosphorylation level of AMPKα was observed in RZM-treated HCC cells than those of Met. Simultaneously, increased protein levels of p21 were attenuated by AMPK specific inhibitor compound C.

## Conclusions

In conclusion, our study reveals that RZM has an inhibitory function on HCC cells growth by upregulating p21 in an AMPK-dependent manner, and may be a novel candidate jointly applied in HCC therapy.

## Additional files

**Additional file 1: Figure S1.** Representative images of hoechst staining revealed the nuclear morphology of HepG2 cells with indicated treatment. (200×; scale bar, 100 μm).

**Additional file 2: Figure S2.** Weight curves of the nude mice were analyzed.

## Abbreviations

RZM: rosiglitazone metformin adduct
Ros: rosiglitazone
Met: metformin
HCC: hepatocellular carcinoma
AMPK: AMP-activated protein kinase
DM: diabetes mellitus
T2DM: type 2 diabetes mellitus
CDKN1A: cyclin dependent kinase inhibitor 1A
