# Supplementary figures and images for: Rosiglitazone metformin adduct inhibits hepatocellular carcinoma proliferation via activation of AMPK/p21 pathway
Source: Cancer Cell Int. 2019 Jan 11;19:13. doi: 10.1186/s12935-019-0732-2 (PMC6330460; doi:10.1186/s12935-019-0732-2)

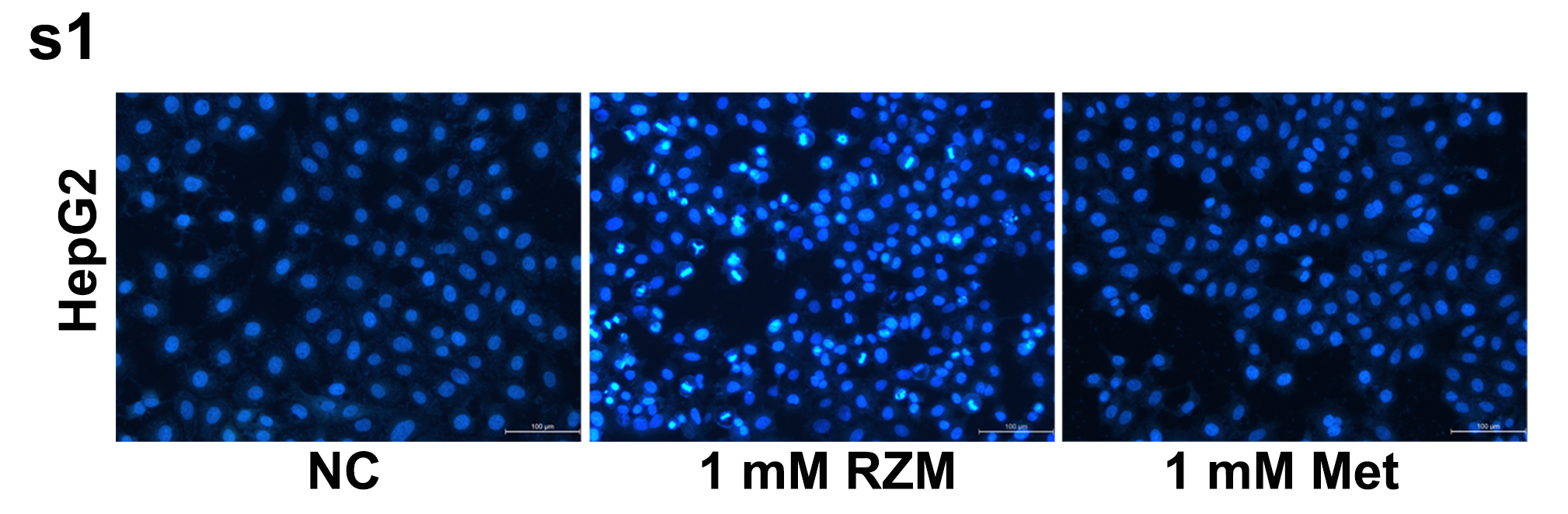

Supplement: Supplementary file 1 — Additional file 1: Figure S1. Representative images of hoechst staining revealed the nuclear morphology of HepG2 cells with indicated treatment. (200×; scale bar, 100 μm). [file 12935_2019_732_MOESM1_ESM.tif]

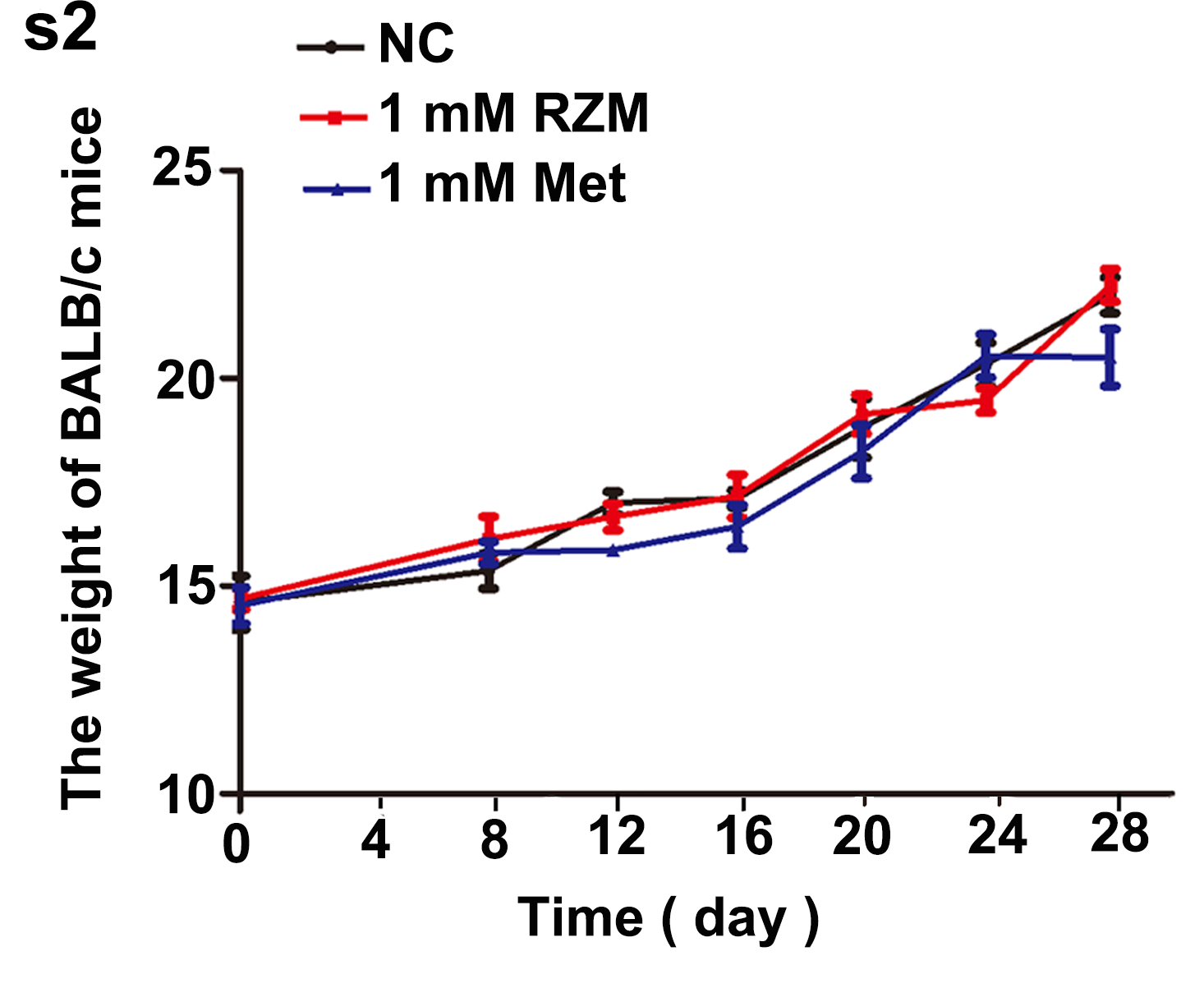

Supplement: Supplementary file 2 — Additional file 2: Figure S2. Weight curves of the nude mice were analyzed. [file 12935_2019_732_MOESM2_ESM.tif]
